# Moderate Folic Acid Supplementation in Pregnant Mice Results in Behavioral Alterations in Offspring with Sex-Specific Changes in Methyl Metabolism

**DOI:** 10.3390/nu12061716

**Published:** 2020-06-08

**Authors:** Marta Cosín-Tomás, Yan Luan, Daniel Leclerc, Olga V. Malysheva, Nidia Lauzon, Renata H. Bahous, Karen E. Christensen, Marie A. Caudill, Rima Rozen

**Affiliations:** 1 Departments of Human Genetics and Pediatrics, Research Institute of the McGill University Health Center, McGill University, Montreal, QC H4A 3J1, Canada; marta.cosin@isglobal.org (M.C.-T.); yan.luan@mail.mcgill.ca (Y.L.); daniel.leclerc@mail.mcgill.ca (D.L.); renatabahous@gmail.com (R.H.B.); karen.christensen@mail.mcgill.ca (K.E.C.); 2 Division of Nutritional Sciences and Genomics, Cornell University, Ithaca, NY 14853, USA; ovm4@cornell.edu (O.V.M.); mac379@cornell.edu (M.A.C.); 3 Drug Discovery Platform, Research Institute of the McGill University Health Centre, Montreal, QC H4A 3J1, Canada; nidia.lauzon@muhc.mcgill.ca

**Keywords:** choline, folate, MTHFR, neurodevelopment, phospholipids

## Abstract

Fifteen to 20% of pregnant women may exceed the recommended intake of folic acid (FA) by more than four-fold. This excess could compromise neurocognitive and motor development in offspring. Here, we explored the impact of an FA-supplemented diet (5× FASD, containing five-fold higher FA than recommended) during pregnancy on brain function in murine offspring, and elucidated mechanistic changes. We placed female C57BL/6 mice for one month on control diets or 5× FASD before mating. Diets were maintained throughout pregnancy and lactation. Behavioural tests were conducted on 3-week-old pups. Pups and mothers were sacrificed at weaning. Brains and livers were collected to examine choline/methyl metabolites and immunoreactive methylenetetrahydrofolate reductase (MTHFR). 5× FASD led to hyperactivity-like behavior and memory impairment in 3-week-old pups of both sexes. Reduced MTHFR protein in the livers of FASD mothers and male pups resulted in choline/methyl metabolite disruptions in offspring liver (decreased betaine) and brain (decreased glycerophosphocholine and sphingomyelin in male pups, and decreased phosphatidylcholine in both sexes). These results indicate that moderate folate supplementation downregulates MTHFR and alters choline/methyl metabolism, contributing to neurobehavioral alterations. Our findings support the negative impact of high FA on brain development, and may lead to improved guidelines on optimal folate levels during pregnancy.

## 1. Introduction

Folate is essential for optimal brain function since it is required for methylation reactions, synthesis of nucleotides, neurotransmitters and myelin, and maintenance of homocysteine at non-toxic levels [1]. Low intake of folate during pregnancy and a common genetic variant in folate metabolism (methylenetetrahydrofolate reductase (MTHFR), 677C > T, present at ~15% homozygosity in Caucasians) are associated with an increased risk of neural tube defects (NTD) and other neurodevelopmental disorders (ND) such as autism spectrum disorder (ASD) and schizophrenia [2,3]. Therefore, women are encouraged to consume supplements of folic acid (FA; synthetic form of folate) at ≥400 µg/day periconceptionally [4]. Food fortification with FA is now routine in many countries and has reduced NTD rates [4]. However, recent concerns have been raised regarding high folate intake during pregnancy and lactation, since it has been estimated that 15–20% of pregnant women exceed recommended levels by more than four-fold due to food fortification and increased use of supplements [4,5,6]. Several epidemiological studies have shown that high FA intake can negatively impact motor, social and neurocognitive development in offspring [5,6]. Animal studies have shown that DNA methylation and gene expression [7,8,9,10,11], brain fatty acid content [12], and neurotrophic factor levels [13] are altered in offspring brain by maternal FA supplementation. We recently identified a novel mechanism by which high folate may be deleterious: reduced hepatic MTHFR protein [14,15]. In pregnant mice consuming folate at levels 10-fold higher than recommended (10× FASD), we observed this pseudo-MTHFR deficiency in the liver of dams and their male pups. These male pups had disturbances in methyl metabolism and memory impairment [15]. MTHFR is a ubiquitous enzyme that generates 5-methyltetrahydrofolate for the remethylation of homocysteine to methionine, the precursor of S-adenosylmethionine (SAM), a major methyl donor. MTHFR deficiency is associated with reduced circulatory folate and reduced methylation potential. When folate metabolism is disturbed, choline can be used as an alternate methyl donor through generation of betaine, which donates its methyl group for methionine synthesis, thus facilitating SAM synthesis in a folate-independent manner. When folate metabolism is compromised, the alternate pathway is upregulated, affecting choline pools [16,17]. Choline is the precursor for many important phospholipids and for the neurotransmitter acetylcholine (Acho) [17]. Altered levels of these choline-derived metabolites are associated with cognitive impairment, attention deficits and ASD [18,19,20]. Our rodent studies and those of other groups have been informative regarding potential mechanisms, but the studies used diets with folate concentrations that were at least 10-fold higher than recommended [8,10,15,21], or had other micronutrient deficiencies [9,12,13], or the diets were maintained post-weaning [7]. Furthermore, few studies included female offspring even though sex differences are apparent across multiple NDs [22]. The goal of this study was to determine whether a more moderate folate supplementation (5× FASD) affects brain function in both male and female offspring at the same developmental stage as in our previous study using 10× FASD [15] and to identify potential mechanisms. Our findings indicate that moderately increased folate intake in pregnancy downregulates MTHFR and alters choline/methyl metabolism, contributing to neurobehavioral alterations in pups.

## 2. Materials and Methods

### 2.1. Animals and Dosage Information

Animal experimentation was performed according to the guidelines of the Canadian Council on Animal Care and approved by the Animal Care Committee of the Research Institute of the McGill University Health Center (AUP 3132). Mice were housed in specific-pathogen-free facilities at 18–24 °C with a 12 h light/dark cycle, and water and food ad libitum. At weaning, female C57BL/6 mice were randomly placed on amino acid defined Control Diet (CD) (2 mg FA/kg diet, recommended level for rodents) [23] (TD.01369, Harlan Laboratories, Inc., Madison, WI, USA) or Folic Acid Supplemented Diet (FASD), which contained five times the amount of FA recommended for rodents (10 mg FA/kg diet, TD.08278, Harlan Laboratories, Inc., Madison, WI, USA) (Table S1). Both diets contained 2.5 g/kg choline bitartrate, 3.3 g/kg L-methionine and 1% of an antibiotic that inhibits folate synthesis by intestinal flora, succinylsulfathiazole. The antibiotic is used to reduce experimental variability, to establish an accurate/known level of folate availability, and for continuity with our previous work. Five to six weeks after starting diets, females were mated with C57BL/6 males. Diets were maintained throughout pregnancy and lactation. We conducted behavioral testing on offspring at postnatal day (pd) 19, 20, 25 and 27. Mothers and offspring were sacrificed at weaning (4 weeks of age for pups) by CO_2_ asphyxiation in random order, and body weights were measured. Maternal and 4-week-old pup livers and pup cerebral cortices were collected, snap frozen and stored at −80 °C. Whole cortices were dissected based on visual information, such as differences in color of adjacent tissues, and on the natural anatomical boundaries (e.g., using midline and cerebellum as landmarks), as described in Spijker et al. [24]. For some animals, brains were cut in half and frozen intact for analysis with MALDI-IMS.

### 2.2. Behavioral Testing

Mice were subjected to three behavioral tests (open field test (OF) at pd 19, novel object recognition test (NOR) at pd 20, and reciprocal social interaction test at pd 25), as well as grip strength measurements at pd 27, as previously performed [25,26,27]. Animals rested for 5 days between NOR test and social interaction test (OF test corresponded to the habituation day of the NOR). Animals that conducted social interaction tests were not used for grip strength measurements because of temporal proximity between assessments. Tests were always performed before 12 pm. The testing room was normally illuminated and kept at a similar temperature to the colony room. Animals were transferred to the room 30 min. before starting the test. OF, NOR and reciprocal social interaction tests were videotaped, and scored by a blinded investigator after the sessions.

#### 2.2.1. OF Test

OF test assesses anxiety-related and exploratory behavior. As in previous studies [25], mice were placed in the middle of an OF box (opaque gray 18” × 18” × 18” Plexiglas) and movements videotaped for 5 min. Sessions were analyzed using Any-Maze software (Stoelting Co., Wood Dale, IL, USA) to measure distance, speed, and movement pattern. Time spent self-grooming and rearing was also analyzed. Anxiety index is calculated as distance traveled in four central squares relative to total distance traveled [28].

#### 2.2.2. NOR Test

NOR test measures cognitive abilities, in particular short-term memory. As previously performed [25], 24 h prior to the test, mice were habituated to the apparatus (opaque gray 18” × 18” × 18” Plexiglas box) for 10 min. The first 5 min of this habituation phase were recorded and constituted the OF test (explained above). The day after, animals were placed alone in the same box and engaged to explore two identical copies of an object (object 1) for a 10 min training session. Sixty min after the training session, animals were placed alone in the same box for a 5 min test trial. During the test trial, animals explored a familiar object (object 1) and a novel object (object 2). The test session was recorded and the amount of time exploring objects was measured using Any-Maze. The discrimination index (DI) is the amount of time animals spent exploring the novel object minus time spent exploring the familiar object divided by time spent exploring both familiar and novel objects. Since this test is based on the spontaneous tendency of rodents to spend more time exploring a novel object than a familiar one, a negative value indicates that the animal shows no or less preference for a novel object, probably due to memory impairment.

#### 2.2.3. Reciprocal Social Interactions

In the social interaction test, exploratory behavior towards an unfamiliar mouse is studied. As before [26], there were two habituation sessions, in which the mouse was placed alone in the testing box (opaque gray 18” × 18” × 18” Plexiglas box) to habituate for 10 min. The day of testing, a pair of unfamiliar pups of the same sex, age and condition (same diet, but socially naive to each other prior to the social test session-i.e., from different litters) were placed in the same testing box. The two unfamiliar juvenile mice were engaged in social interaction for 5 min. The test session was recorded. The evaluated parameters included number of social contacts, mean duration of contacts, total social interaction time, frequency of contacts, duration of the longest contact, latency to first contact, and were scored from recordings with Any-Maze.

#### 2.2.4. Grip Strength Measurement

The grip strength meter allows the measurement of neuromuscular function as maximal muscle strength of forelimbs, by the grasping of a grid connected to a sensor (Harvard Apparatus, Holliston, MA, USA). As previously performed [27], the investigator lowered the mouse over the grid, keeping the torso horizontal and allowing only its forepaws to attach to the grid before any measurements were taken, then gently pulled the mouse back by its tail, ensuring the mouse gripped the top portion of the grid and the torso remained horizontal. The maximal grip strength value displayed on the screen was recorded. This procedure was repeated nine times at one-min. intervals to obtain a mean value for the nine forelimb measurements. After the test, the animal was weighed to normalize grip strength values against body weight. Trials were excluded if only one forepaw or hindlimbs were used and if the mouse turned during the pull or left the bar.

### 2.3. Western Blotting

Immunoblotting of protein extracts from cortex and liver was conducted as before [14]. Primary antibodies were MTHFR [29] and Vinculin (Cell Signaling Technology, Boston, MA, USA). Secondary antibody was horseradish peroxidase (HRP)-conjugated donkey anti-rabbit IgG (GE Healthcare, Mississauga, Canada). Detection was achieved using ECL Prime Western Blotting Detection Reagent (GE Healthcare, Mississauga, ON, Canada). Bands were quantified by densitometry using Amersham Imager 600 Analysis Software v.1.0.0 (GE Healthcare, Mississauga, Canada) and normalized to Vinculin.

### 2.4. Measurement of SAM, SAH and Choline Metabolites by Liquid Chromatography-Electrospray Ionization Tandem Mass Spectrometry (LC-MS)

Choline, betaine, glycerophosphocholine (GPC), phosphatidylcholine (PtdCho), sphingomyelin (SM), and phosphocholine (PCho) were measured in the cortex and liver according to protocols previously published with modifications [30,31,32]. Lysophosphatidylcholine (LPC) and acetylcholine (Acho) were measured in liver or cortex, respectively. SAM and SAH concentrations were determined by LC-MS (Thermo Fisher Scientific, San Jose, CA, USA) with modifications based on instrumentation [33].

### 2.5. Measurement of PtdCho and SM by Matrix-Assisted Laser Desorption Ionization Imaging Mass Spectrometry (MALDI-IMS)

#### 2.5.1. Sample Preparation

Right sagittal brains were cut at 10 µm thickness using a Leica CM1950 cryostat (Leica Microsystems GmbH, Wetzlar, Germany) to a depth of 1400 μm and thaw-mounted on an ITO-coated microscope glass slide (Delta Technologies Ltd., Loveland, CO, USA). Upon desiccation, the MALDI matrix 1, 5-diaminonaphtalene (1, 5-DAN) was deposited onto the tissue sections using the HTX M3 TM-sprayer (HTX Technologies, Chapel Hill, NC, USA).

#### 2.5.2. Imaging Mass Spectrometry (IMS)

IMS of sections was performed on a MALDI TOF/TOF Ultraflextreme mass spectrometer (Bruker Daltonics, Billerica, MA, USA). IMS data were acquired in positive mode with a spatial resolution of 100 μm and visualized using the software SCiLS (2019 b Premium 3D, Bremen, Germany). Lipid identification was confirmed by MS/MS and using the database LIPID MAPS^®^ Lipidomics Gateway. Available online: https://www.lipidmaps.org/ (accessed on 27/02/2019).

### 2.6. Statistical Analysis

Maternal body weight and consumed diet were analyzed using linear mixed models (MIXED procedure, SPSS Statistics version 20; IBM, Armonk, NY, USA) by specifying maternal diet as fixed effect, and including the size of the litter as a random effect. Offspring body weight and behavioral testing results were analyzed using linear mixed models (MIXED procedure, SPSS Statistics version 20; IBM) by specifying maternal diet, offspring sex, and maternal diet x offspring sex interaction as fixed effects, and including litter as a random effect. Since only one animal per litter was used for metabolite and protein-expression quantification experiments, these data were analyzed by 2-factor ANOVA (containing maternal diet and offspring sex as factors) followed by post hoc analysis corrected for multiple testing with Tukey’s procedure, unless indicated. Litter size and protein expression in lactating mothers were analyzed using unpaired *t*-test. Pearson’s correlations were conducted to analyze the association between NOR DI and OF total active time or metabolite concentration. ANOVA, *t*-test, and correlations were performed with GraphPad Prism software (version 6.01; GraphPad Software, San Diego, CA, USA). Individual mice were used as the unit of analysis for all calculations. Statistical outliers determined by Grubbs’ test were removed from the analyses. For all analyses, *p* < 0.05 was considered significant. All data are presented as means ± SEM.

## 3. Results

### 3.1. 5× FASD during Pregnancy and Lactation Leads to Behavioral Alterations in Offspring

Female C57BL/6 mice were placed on CD or 5× FASD pre-pregnancy (at weaning) for one month, and continued throughout pregnancy and lactation. CD and FASD mothers consumed similar amounts of food (Diet *p* = 0.165, *n* = 13–16/group), and had similar litter sizes (Diet *p* = 0.608, *n* = 23–25/group). There were no differences in maternal or in 4-week-old offspring body weights between groups (CD mothers: 25.32 ± 0.3 g, FASD mothers: 26.03 ± 0.4 g, Diet *p* = 0.1, *n* = 19–20/group; CD male pups: 14.69 ± 0.1 g, FASD male pups: 15.59 ± 0.14 g, CD female pups: 13.29 ± 0.13 g, FASD female pups: 14.02 ± 0.17 g, Diet *p* = 0.1, *n* = 25–33/group).

Offspring were assessed for behavior and motor function in the following order: OF test for general locomotor activity levels and anxiety at pd 19, NOR test for short-term memory at pd 20, and social interaction test at pd 25 for social behavior. Grip strength was measured at pd 27 to assess neuromuscular function.

**Figure 1:**
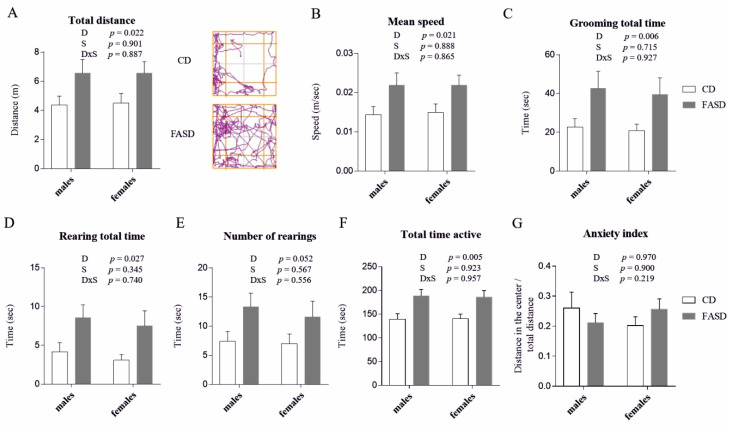
Open field (OF) test in 3-week-old male and female offspring. (**A**) FASD pups traveled greater distances than CD pups (left panel). Representative tracking plots for a CD and a FASD animal are shown (right panel). (**B**) FASD pups traveled at higher mean speeds than CD pups. (**C**) FASD pups spent more time grooming than CD pups. (**D**) FASD pups spent more time rearing than CD pups. (**E**) FASD pups had more rearing episodes than CD pups, although the difference did not reach statistical significance. (**F**) FASD pups spent more total time active (i.e., grooming, rearing and traveling) than CD pups. (**G**) There were no differences in the anxiety index. Anxiety index is calculated as distance traveled in the 4 center squares divided by total distance traveled. *n* = 15–17/group, 10–11 litters/diet. White bars: CD animals, gray bars: FASD animals. Values are means ± SEM. *p* values from linear mixed-model analysis (including maternal diet and offspring sex as fixed factors and litter as a random factor) are indicated at the top of each graph. CD: Control diet, FASD: Folic acid supplemented diet, D: Diet, S: Sex, D×S: Diet × Sex interaction.

Three-week-old FASD pups showed hyperactivity-like behavior compared to CD mice. They traveled greater distances within the OF box during the 5-min. test (Figure 1A, Diet *p* = 0.022) and with higher speeds (Figure 1B, Diet *p* = 0.021). They spent more time grooming (Figure 1C, Diet *p* = 0.006) and rearing (Figure 1D, Diet *p* = 0.027), and had more rearing episodes, although the latter result was non-significant (Figure 1E, Diet *p* = 0.052). Overall, FASD pups spent more total time active than CD pups (total time active is the sum of the time spent grooming, rearing and traveling) (Figure 1F, Diet *p* = 0.005). This hyperactivity-like behavior is not related to differences in anxiety (anxiety index was calculated as distance traveled in center squares divided by total distance traveled (Figure 1G, Diet *p* = 0.970). Time spent in the center areas was also measured to confirm the lack of anxiety differences; there were no significant differences between the two dietary groups (*p* = 0.243, data not shown).

**Figure 2:**
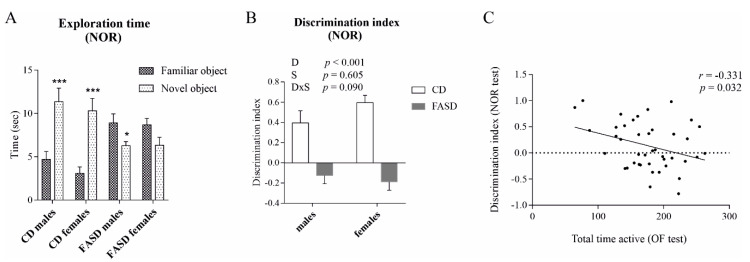
NOR test in 3-week-old male and female offspring. (**A**) CD pups spent more time exploring the novel object compared to the familiar object, whereas FASD pups spent the same or less time exploring the novel object compared to the familiar object. (**B**) Short-term memory impairment, indicated by the negative DI, was observed due to FASD during the NOR test. A negative DI (calculated as time spent exploring novel object minus time spent exploring familiar object divided by total time exploring both novel and familiar objects) indicates that less time was spent with the novel object compared to the familiar object. White bars: CD animals, gray bars: FASD animals. (**C**) DI negatively correlated with total activity time in the OF test. *n* = 10–13/group, 9–11 litters/diet. Values are means ± SEM. *p* values from linear mixed-model analysis (including maternal diet and offspring sex as fixed factors and litter as a random factor) are indicated at the top of each graph. * *p* < 0.05, *** *p* < 0.001 (*t*-test). CD: Control diet, FASD: Folic acid supplemented diet, D: Diet, S: Sex, D×S: Diet × Sex interaction, *r*: Pearson’s correlation coefficient.

In the NOR test, CD pups spent more time exploring the novel object, as expected for control mice, whereas FASD pups spent less time exploring the novel object (Figure 2A, time exploring novel vs. familiar object: CD males *p* = 0.001; CD females *p* < 0.001; FASD males *p* = 0.039; FASD females *p* = 0.06). FASD pups demonstrated a negative DI, whereas CD pups demonstrated a positive value (Figure 2B, Diet *p* < 0.001). There was no significant sex effect or interaction for any parameter. Notably, DI negatively correlated with total activity time in the OF test, suggesting that hyperactive mice performed more poorly in the memory test (Figure 2C, *r* = −0.331, *p* = 0.032).

There were no differences due to diet or sex for grip strength (Diet *p* = 0.934; Sex *p* = 0.184; Diet × Sex *p* = 0.415), or social interaction (time spent interacting with a non-sibling equal, Diet *p* = 0.962, Sex *p* = 0.656, Diet × Sex *p* = 0.148; number of social contacts, Diet *p* = 0.856, Sex *p* = 0.706, Diet × Sex *p* = 0.107) (Figure S1).

These findings suggest that maternal 5× FASD during pregnancy and lactation leads to hyperactivity-like behavior and short-term memory impairment in offspring of both sexes.

### 3.2. Reduced MTHFR Protein in Maternal and Male Offspring Liver

**Figure 3:**
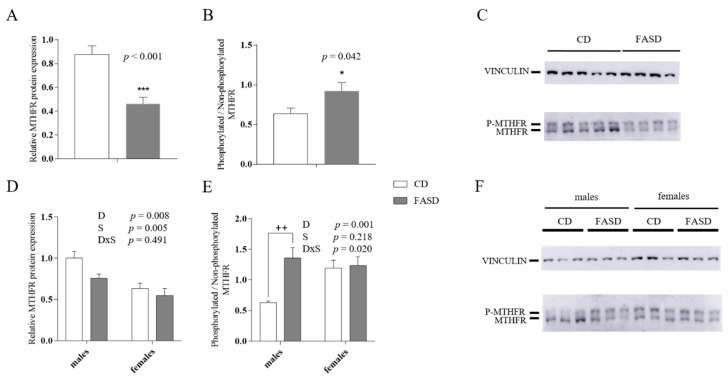
MTHFR protein in maternal and offspring liver. (**A**) Total immunoreactive MTHFR protein (normalized to vinculin expression) was reduced in livers of FASD mothers (*n* = 9–10/group). (**B**) The ratio of the phosphorylated isoform (less active) to the non-phosphorylated isoform was increased in FASD mothers (*n* = 10/group). (**C**) Representative Western blot of liver extracts from CD and FASD mothers. (**D**) Total immunoreactive MTHFR protein (normalized to vinculin expression) was reduced in livers of FASD pups compared to CD pups, and in female pups compared to male pups (*n* = 5–7/group). (**E**) The ratio of the phosphorylated isoform to the non-phosphorylated isoform was increased in FASD male pups (*n* = 5–7/group). (**F**) Representative Western blot of liver extracts from 4-week-old male and female pups. White bars: CD animals, gray bars: FASD animals. Values are means ± SEM. In graphs (**A**,**B**), asterisks correspond to *t*-test associated *p* values, * *p* < 0.05, *** *p* < 0.001. In graphs (**C**,**D**), *p* values from 2-factor ANOVA are indicated at the top of each graph, and Tukey post-hoc significant *p* values are indicated as ++*p* < 0.01. CD: Control diet, FASD: Folic acid supplemented diet, D: Diet, S: Sex, D×S: Diet × Sex interaction, P-MTHFR, phosphorylated MTHFR isoform.

Since we had previously shown that 10× FASD triggered a pseudo-MTHFR deficiency in murine liver, we measured MTHFR protein in livers of mothers and offspring. Hepatic MTHFR protein expression was reduced in FASD mothers (*p* < 0.001, *t*-test) (Figure 3A,C). In addition, the ratio of phosphorylated to non-phosphorylated MTHFR was increased due to FASD (*p* = 0.042, *t*-test) (Figure 3B,C). The phosphorylated form of MTHFR is the less active form of the enzyme [34].

In pups, there was also a reduction in total MTHFR protein due to FASD, although this response was seen primarily in males (Diet *p* = 0.008; Tukey’s post-hoc CD vs. FASD males: *p* = 0.082; CD vs. FASD females: *p* = 0.455) (Figure 3D,F). Females showed lower levels of MTHFR than males regardless of diet (Sex *p* = 0.005) (Figure 3D,F). The ratio of phosphorylated to non-phosphorylated MTHFR was increased due to FASD only in male offspring (Diet *p* = 0.001, Diet × Sex *p* = 0.02; Tukey’s post-hoc CD vs. FASD males: *p* = 0.001; CD vs. FASD females: *p* = 0.88) (Figure 3E,F).

There were no differences in total MTHFR protein nor in the phosphorylated to non-phosphorylated ratio between groups in the cortex (total MTHFR: Diet *p* = 0.905, Sex *p* = 0.810, Diet × Sex *p* = 0.359; phosphorylated to non-phosphorylated MTHFR ratio: Diet *p* = 0.109, Sex *p* = 0.151, Diet x Sex *p* = 0.077) (data not shown).

Thus, FASD during pregnancy and lactation leads to pseudo-MTHFR deficiency in maternal and offspring liver, with an effect primarily in male offspring.

### 3.3. Altered Concentration of Choline-Derived Metabolites in Liver of FASD Offspring

**Figure 4:**
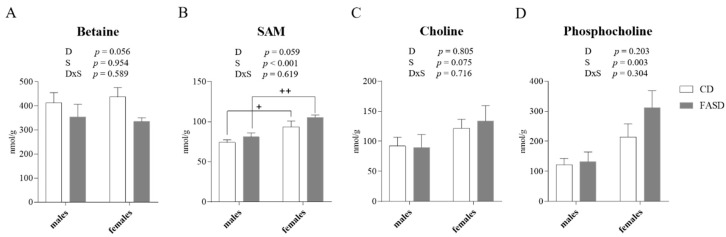
Concentrations of choline-derived metabolites measured by LC-MS in offspring liver. (**A**) FASD pups showed reduced betaine compared to CD pups. (**B**) FASD pups showed increased SAM compared to CD pups, and male pups showed reduced SAM compared to females. (**C**) Male pups showed reduced choline compared to females. (**D**) Male pups showed reduced PCho compared to females. *n* = 7–8/group. White bars: CD animals, gray bars: FASD animals. Values represent means ± SEM. *p* values from 2-factor ANOVA are indicated at the top of each graph, and Tukey post-hoc significant *p* values are indicated as +*p* < 0.05, ++*p* < 0.01. CD: Control diet, FASD: Folic acid supplemented diet, D: Diet, S: Sex, D×S: Diet × Sex interaction.

Previous studies in our lab and others have shown that folate disturbances can trigger changes in choline metabolism, since these two pathways intersect in the provision of methyl donors for synthesis of methionine/SAM. Choline can be converted to betaine, a folate-independent methyl donor for homocysteine remethylation to methionine [17]. Therefore, we measured SAM, SAH, methionine, and choline-derived metabolites (betaine, choline, GPC, LPC, PCho, PtdCho, and SM) by LC-MS in offspring liver. Although the differences did not reach statistical significance, betaine concentration tended to be lower in FASD (Diet *p* = 0.056, Figure 4A), and SAM tended to be higher in FASD (Diet *p* = 0.059, Figure 4B). Although non-significant (*p* = 0.059), the change in betaine reflects increased use of the alternate pathway to compensate for reduced MTHFR, or possibly other disturbances related to high folate intake. Male offspring showed reduced concentrations of SAM, choline, and PCho compared to females irrespective of diet (Sex *p* < 0.001, *p* = 0.075, *p* = 0.003, respectively, Figure 4B–D). This observation suggests that females may be more efficient in maintaining SAM concentrations, and is consistent with earlier work in which we suggested that males rely more on the folate-independent methyl-donor pathway than females [35]. There were no differences by diet or sex for the other metabolites (Figure S2).

### 3.4. Altered Concentration of Choline-Derived Metabolites in Cerebral Cortex of FASD Offspring

**Figure 5:**
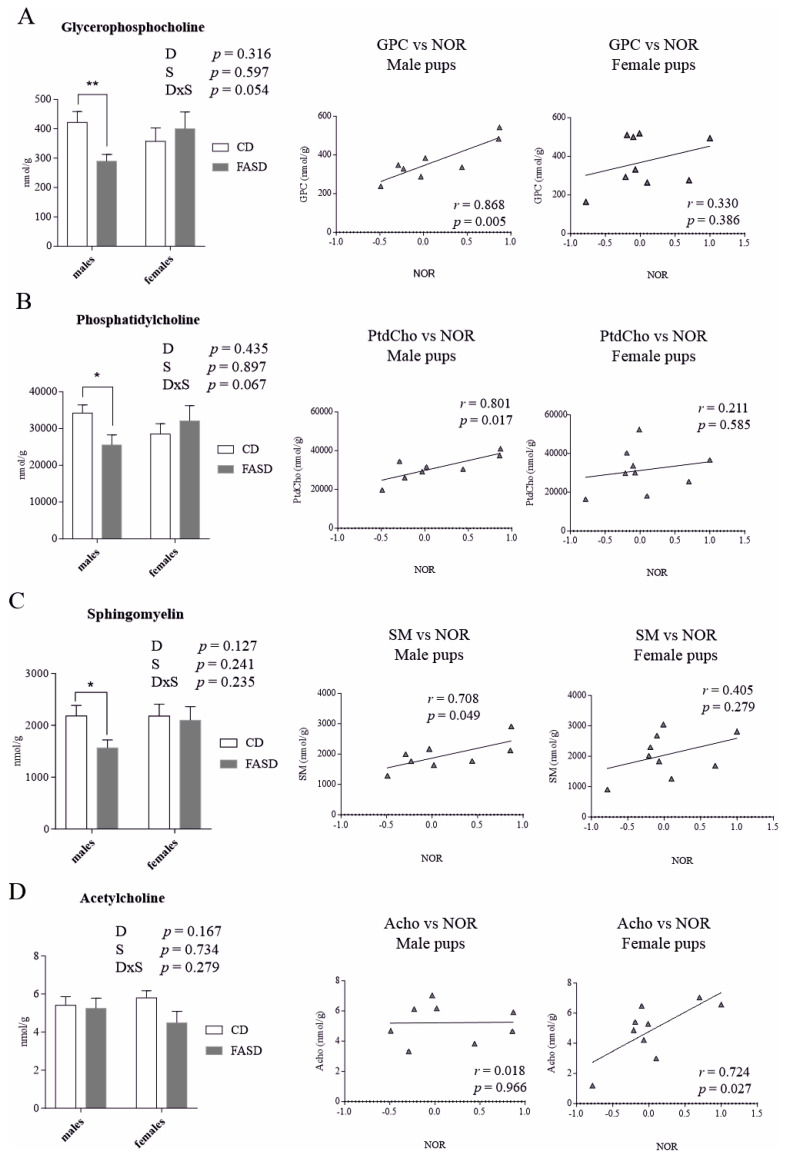
Concentrations of choline-derived metabolites measured by LC-MS in offspring cerebral cortex. (**A**) FASD male pups showed a reduction in GPC compared to CD male pups; females did not show dietary changes. GPC concentration in male pups positively correlated with the DI in the NOR test; female pups did not show a significant correlation. (**B**) FASD male pups showed a reduction in PtdCho compared to CD males; females did not show dietary changes. PtdCho levels in male pups positively correlated with the DI in the NOR test; female pups did not. (**C**) FASD male pups showed a reduction in SM compared to CD males; females did not. SM concentration in male pups positively correlated with the DI in NOR test; female pups did not show this correlation. (**D**) Acho concentrations in female pups positively correlated with the DI in NOR test; male pups did not show this correlation. *n* = 7–8/group. White bars: CD animals, gray bars: FASD animals. Bar graphs represent means ± SEM. *p* values from 2-factor ANOVA are indicated at the top of each graph. Asterisks correspond to *t* test associated *p* values, indicated as * *p* < 0.05, ** *p* < 0.01. CD: Control diet, FASD: Folic acid supplemented diet, D: Diet, S: Sex, D×S: Diet × Sex interaction, *r*: Pearson’s correlation coefficient.

The liver is the key organ for one-carbon metabolism. Metabolic alterations in liver can alter circulatory metabolites and impact other tissues such as brain [36]. Consequently, we measured SAM, SAH, methionine, and choline-derived metabolites (acetylcholine (Acho), betaine, choline, GPC, PCho, PtdCho, and SM) by LC-MS in offspring cortex. There were no significant diet or sex effects for any of the metabolites. Differences between groups were not significant by Tukey’s post-hoc. However, when data were examined separately by sex, we observed that male pups, and not female pups, showed a significant reduction in GPC, PtdCho, and SM by *t* test *p* = 0.008, *p* = 0.036, *p* = 0.03, respectively) (Figure 5A–C, left panels).

Of interest is the fact that male pups showed a positive correlation between these three metabolites and the NOR results (GPC vs. DI in males: *r* = 0.868, *p* = 0.005, PtdCho vs. DI in males: *r* = 0.801, *p* = 0.017, SM vs. DI in males: *r* = 0.708, *p* = 0.049) (Figure 5A–C, middle panels). Female pups, which did not show dietary differences for these three metabolites, did not show significant correlations (Figure 5A–C, right panels). GPC is formed in the breakdown of PtdCho and along with PCho constitutes a major form of choline storage in cytosol. PtdCho is a major component of membranes and affects membrane integrity. SM is a major component of myelin [37].

In females, there was a significant correlation between Acho concentration and NOR results (*r* = 0.724, *p* = 0.027); this correlation was not observed in males (Figure 5D, middle and right panels). Acho is involved in memory, arousal and attention [38].

**Figure 6:**
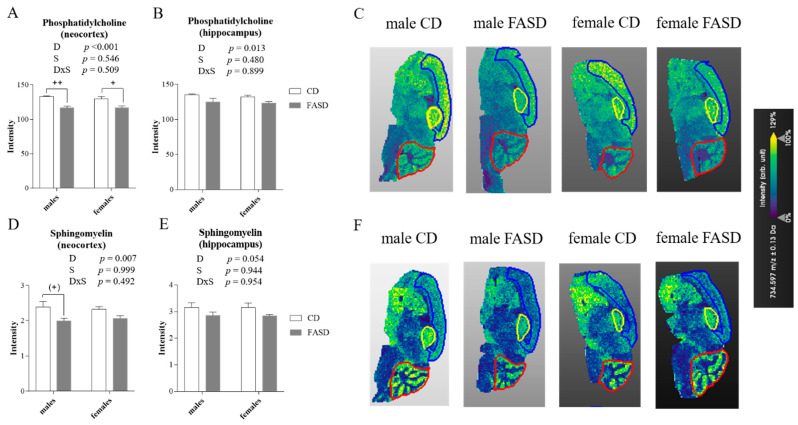
PtdCho and SM measured by MALDI-IMS in offspring brain. (**A**) The two most abundant species of PtdCho (m/z 734 + 760) were decreased in neocortex of FASD pups. (**B**) These 2 species were also decreased in hippocampus of FASD pups (**C**) Representative mapping of the different signal intensities (arbitrary units) for PtdCho (m/z 734) in neocortex (designated by a blue line), hippocampus (yellow line), and cerebellum (red line). (**D**) SM was decreased due to FASD in neocortex, predominantly in males. (**E**) SM was decreased due to FASD in hippocampus although this change was not statistically significant. (**F**) Representative mapping of the different signal intensities (arbitrary units) for SM in neocortex (designated by a blue line), hippocampus (yellow line), and cerebellum (red line). *n* = 4/group. White bars: CD animals, gray bars: FASD animals. Bar graphs represent means ± SEM. *p* values from 2-factor ANOVA are indicated at the top of each graph. Asterisks correspond to Tukey post-hoc *p* values, indicated as (+) *p* = 0.064, +*p* < 0.05, ++*p* < 0.01. CD: Control diet, FASD: Folic acid supplemented diet, D: Diet, S: Sex, D×S: Diet × Sex interaction.

The distribution pattern and fatty acid content of phospholipid species in brain reflects regional differences and specialized functions of various regions. Changes in these species can be associated with CNS pathologies [39,40]. To assess the region-specific effects of FASD, we measured PtdCho and SM using MALDI-IMS in pup brains (Figure 6). MALDI-IMS couples lipidomic profiling with in situ localization in biological samples, resulting in the generation of molecular-histological maps from the localization and identification of lipid biomolecules based on mass-to-charge ratio (m/z) [41]. Interestingly, there was a decrease due to diet in the two most abundant species of PtdCho in neocortex (Diet *p* < 0.001) (Figure 6A,C). These two species consist of PtdCho molecules in which the acyl groups contain 32 to 34 carbons with 0 or 1 double bond (m/z 734: PC (32:0) and m/z 760: PC (34:1)). In the hippocampus, we also detected a decrease in these two species due to FASD (Diet *p* = 0.013) (Figure 6B,C). Similarly, SM levels were decreased in neocortex of FASD pups (Diet *p* = 0.007) (Figure 6D,F). Consistent with LC-MS results, the SM reduction was mainly found in male pups (Tukey’s post-hoc: CD vs. FASD males *p* = 0.064; CD vs. FASD females *p* = 0.312) (Figure 6D). In the hippocampus, there was a similar pattern, but the diet effect did not reach statistical significance (Diet *p* = 0.054) (Figure 6E). We did not detect differences between groups for PtdCho or SM in cerebellum (Figure S3).

## 4. Discussion

Epidemiological and animal studies have shown an association between high FA intake during pregnancy and neurobehavioral alterations in offspring. In an earlier murine study, we found memory impairment in male offspring of mothers fed 10× FASD during pregnancy [15]. Reduced MTHFR and choline-methyl metabolism disturbances may have contributed to the cognitive dysfunction. In contrast to previous studies, the current study was designed to assess the effect of a moderate level of FA supplementation. Our study does not distinguish between the effects of high total folate or high FA. However, since we showed that adult mice convert FA readily to THF derivatives with negligible amounts of remaining FA in the liver [14], it is likely that the offspring in this study were not exposed to significant amounts of FA.

Of interest was our finding that pups of both sexes of FASD mothers showed hyperactivity-like behavior. Six independent parameters in the OF test (distance traveled, speed, time grooming or rearing, number of rearing episodes and total time active) were studied and all scores were higher in FASD mice, except for the number of rearing episodes (*p* = 0.052). Since we did not observe changes in anxiety or in neuromuscular function, the hyperactivity cannot be explained by alterations in these parameters. Consistent with these findings, Barua et al. reported hyperactive behavior in adult male offspring of folate-supplemented dams [7]. Valera-Gran et al. found that one-year-old toddlers whose mothers used FA supplements greater than 5000 μg/d during pregnancy scored lower in psychomotor tests [42].

We also observed that FASD offspring had impaired short-term memory, similar to our earlier studies in 10× FASD male pups, although in the current study, we observed this impairment in both sexes. In line with this, Valera-Gran et al. found that FA intake exceeding 1000 μg/d during the periconception window was associated with lower levels of cognitive development in children aged 4–5 years [43]. Given that we measured changes at a stage in which the brain was still developing, studies in older mice would be essential to understand the importance of these changes and to determine whether they are fixed and maintained in adulthood. Low folate in mothers or dams has also been associated with both hyperactivity and memory impairment [44,45], suggesting a U-shaped relationship.

We found a negative correlation between memory and activity. In children with attention deficit hyperactivity disorder (ADHD), hyperactivity is often accompanied by memory impairment [46]. Therefore, the impaired memory in our mice could be caused by the lack of attention associated with hyperactivity. Our finding in FASD mice of phenotypes that are considered features of autistic-like behavior (memory impairment, and repetitive self-grooming [47,48]) is consistent with some epidemiological studies showing a positive association between high maternal folate intake and ASD risk in offspring [49]. As with the aforementioned studies in children, we did not observe sex differences in the murine behavioral response to FASD.

FASD resulted in reduced MTHFR protein in the livers of dams and male pups, as well as an increase in the phosphorylated isoform; these changes would result in MTHFR deficiency. Female pup livers did not show either of these changes. The sex-specific regulation of one-carbon metabolism is not unexpected; we showed that adult male mice utilize the choline/betaine pathway more than the folate-dependent pathway for methionine synthesis [35]. Reduced MTHFR activity leads to increased use of the choline/betaine pathway [16,50] to preserve methyl group homeostasis. Accordingly, we observed a non-significant reduction in betaine (*p* = 0.059) due to FASD in pup livers. However, we saw reductions in this metabolite in both sexes, despite the fact that MTHFR remained stable in females. There may be other regulatory changes in female pups with FASD; additional work is required to identify these changes. The fact that PCho and choline are reduced in males compared to females (significant sex difference for PCho; non-significant for choline, *p* = 0.075) is consistent with the above statement, i.e., males rely more on the folate-independent pathway than females [35]. From earlier work, we know that the MTHFR decreases manifest at earlier times in the pregnancy. Therefore, critical choline/methyl metabolite changes in offspring liver and brain may have begun earlier and may have been more pronounced at earlier stages of development.

During neurodevelopment, the brain’s demand for choline is particularly high; it incorporates PtdCho into membranes and SM into myelin and generates Acho [51]. Reduced betaine in liver suggests that the increased use of choline for betaine-dependent methylation may compromise choline pools/functions. There was a reduction in GPC (a major storage form of choline), PtdCho and SM in cortex of male FASD pups. Circulatory and brain PtdCho levels have been directly associated with cognitive function [18,52], whereas adequate levels of both PtdCho and SM are essential for myelination, a process involved in learning and memory that is altered in ASD and ADHD [53,54]. The positive correlation between cortical levels of these metabolites and the DI in male mice, suggests that reduced levels of these metabolites could be contributing to the altered behavior [15,43,55]. When PtdCho and SM were evaluated by MALDI-IMS, we detected region-specific changes: the two most abundant species of PtdCho and SM were decreased in neocortex and hippocampus of FASD offspring, whereas there were no changes in cerebellum. These changes were more significant in male pups, again alluding to sex differences in reliance on folate-independent methylation. The lack of change in females, again, suggests that other, as-yet unidentified mechanisms may be operating. The fact that behavioral changes were similar between male and female pups even though we observed sex-specific changes in metabolites and MTHFR, suggests that diverse mechanisms influenced by high folic acid consumption may lead to phenotypic convergence. In line with this, it is known that distinct genetic perturbations can lead to phenotypic convergence, especially in the context of complex neuropsychiatric disorders such as ASD [56].

Some of our values did not reach statistical significance and require future studies of larger sample size to demonstrate significant changes. Another issue related to conclusions is the fact that supplementation was initiated when the future mothers were weaned. Therefore, they were exposed to high folate during maturation and before becoming pregnant. It is possible that some of the outcomes are not due solely to direct effects of folate on foetus and neonate, but could relate to endocrine and metabolic alterations, or programming in the mother prior to pregnancy.

In summary, our findings suggest that a moderate FA increase during pregnancy and lactation leads to hyperactivity and memory impairment in the pups of both sexes. The memory changes are consistent with our earlier study using 10× FASD, although we had not examined both sexes [15]. FASD led to a pseudo-MTHFR deficiency in mothers and offspring, producing methyl metabolism disruptions in offspring liver and brain, particularly in male pups. We hypothesize that these perturbations might contribute to the altered behavior through distinct mechanisms in males and females, and that these types of disruptions in one-carbon metabolism may have a role to play in ND such as ADHD or ASD. Our work demonstrates some deleterious consequences of moderate increases of dietary FA on brain function and may lead to improved guidelines on optimal folate levels during pregnancy. The activity of dihydrofolate reductase (DHFR), which converts FA to its active tetrahydrofolate form, is relatively low in humans compared to mice; consequently, increased dietary FA may be even more harmful to humans compared to mice [57]. The negative consequences of high FA in human pregnancies or lactation are therefore more likely to occur at levels of exposure that are commonly achieved through supplementation, fortification and diet.

## Supplementary Materials

The following are available online at https://www.mdpi.com/2072-6643/12/6/1716/s1, Figure S1: Social reciprocal interaction test and grip strength measurements in male and female offspring at pd 25 and pd 27, respectively; Figure S2: Concentrations of choline-derived metabolites and SAH measured by LC-MS in offspring liver; Figure S3: Phosphatidylcholine (PtdCho) and sphingomyelin (SM) measured by MALDI-IMS in offspring cerebellum; Table S1: Composition of Control Diet (CD) and Folic Acid (FASD).
