# Supplementary material for: Moderate Folic Acid Supplementation in Pregnant Mice Results in Behavioral Alterations in Offspring with Sex-Specific Changes in Methyl Metabolism
Source: Nutrients. 2020 Jun 8;12(6):1716. doi: 10.3390/nu12061716 (PMC7352339; doi:10.3390/nu12061716)
Supplement: Supplementary file 1 [file nutrients-12-01716-s001.pdf]

Table S1. Composition of Control Diet (CD) and Folic Acid Supplemented Diet (FASD)[1].

Diet Composition	CD (2mg/kg Folic Acid)[2] TD.01369 (g/kg)	FASD (10mg/kg Folic Acid)[2] TD.08278 (g/kg)
**L-Alanine**	3.5	3.5
**L-Arginine HCl**	12.1	12.1
**L-Asparagine**	6.0	6.0
**L-Aspartic Acid**	3.5	3.5
**L-Cystine**	3.5	3.5
**L-Glutamic Acid**	40.0	40.0
**Glycine**	23.3	23.3
**L-Histidine HCl, monohydrate**	4.5	4.5
**L-Isoleucine**	8.2	8.2
**L-Leucine**	11.1	11.1
**L-Lysine HCl**	18.0	18.0
**L-Methionine**	3.3	3.3
**L-Phenylalanine**	7.5	7.5
**L-Proline**	3.5	3.5
**L-Serine**	3.5	3.5
**L-Threonine**	8.2	8.2
**L-Thryptophan**	1.8	1.8
**L-Tyrosine**	5.0	5.0
**L-Valine**	8.2	8.2
**Sucrose**	349.53	349.422
**Corn Starch**	150.0	150.0
**Maltodextrin**	150.0	150.0
**Soybean Oil**	80.0	80.0
**Cellulose**	30.0	30.0
**Mineral Mix, AIN-93-M-MX**	35.0	35.0
**Calcium Phosphate, monobasic, monohydrate**	8.2	8.2
**Succinylsulfathiazole**	10.0	10.0
**Vitamin Mix, AIN-93-VX**	10.0	10.0
**Choline Bitartrate**	2.5	2.5
**Vitamin K, menadione sodium bisulfite**	0.05	0.05
**tert-Butylhydroquinone (TBHQ), antioxidant**	0.02	0.02
**Folic Acid**	-	0.008
**Red food color**	-	0.1

[1]Diet formulation is based on TD.99366 (a standard amino acid defined diet; Harlan). Vitamin, mineral and choline content is based on recommendations for AIN-93G (Reeves PG. J Nutr 1997; 127:838S-41S); amino acid content is based on Rogers QR and Harper AE. J Nutr 1965; 87:267-73. Methionine content is lower than in Rogers and Harper; however, total methionine + cysteine content exceeds the minimum stated in the NRC guidelines (National Research Council. Nutrient Requirements of Laboratory Animals: 4th ed. Washington, DC: National Academies Press (US), 1995).

[2] Vitamin Mix AIN-93-VX provides a concentration of 2 mg/kg folic acid to the diet.

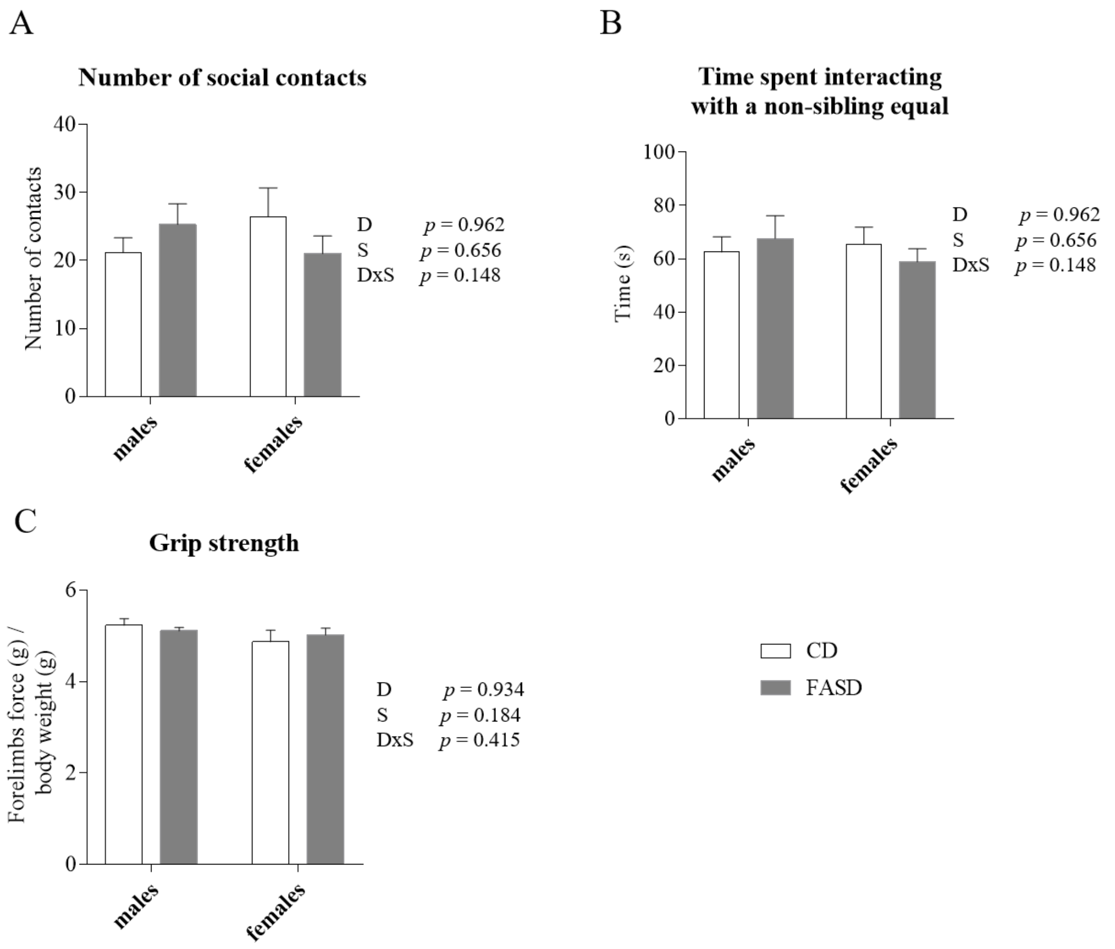

Figure S1. Social reciprocal interaction test and grip strength measurements in male and female offspring at pd 25 and pd 27, respectively.

There were no differences between groups for the number of social contacts (A) or for the time spent interacting with a non-sibling equal (B) during the social reciprocal interaction test. N=13-15 pairs/group, 14-17 litters/diet. (C) There were no differences in grip strength. Grip strength values are normalized against body weight. N=11-12/group, 7-9 litters/diet.

White bars: CD animals, gray bars: FASD animals. Values are means ± SEM. *p* values from linear mixed-model analysis (including maternal diet and offspring sex as fixed factors and litter as a random factor) are indicated at the top of each graph. CD: Control diet, FASD: Folic acid supplemented diet, D: Diet, S: Sex, DxS: Diet x Sex interaction.

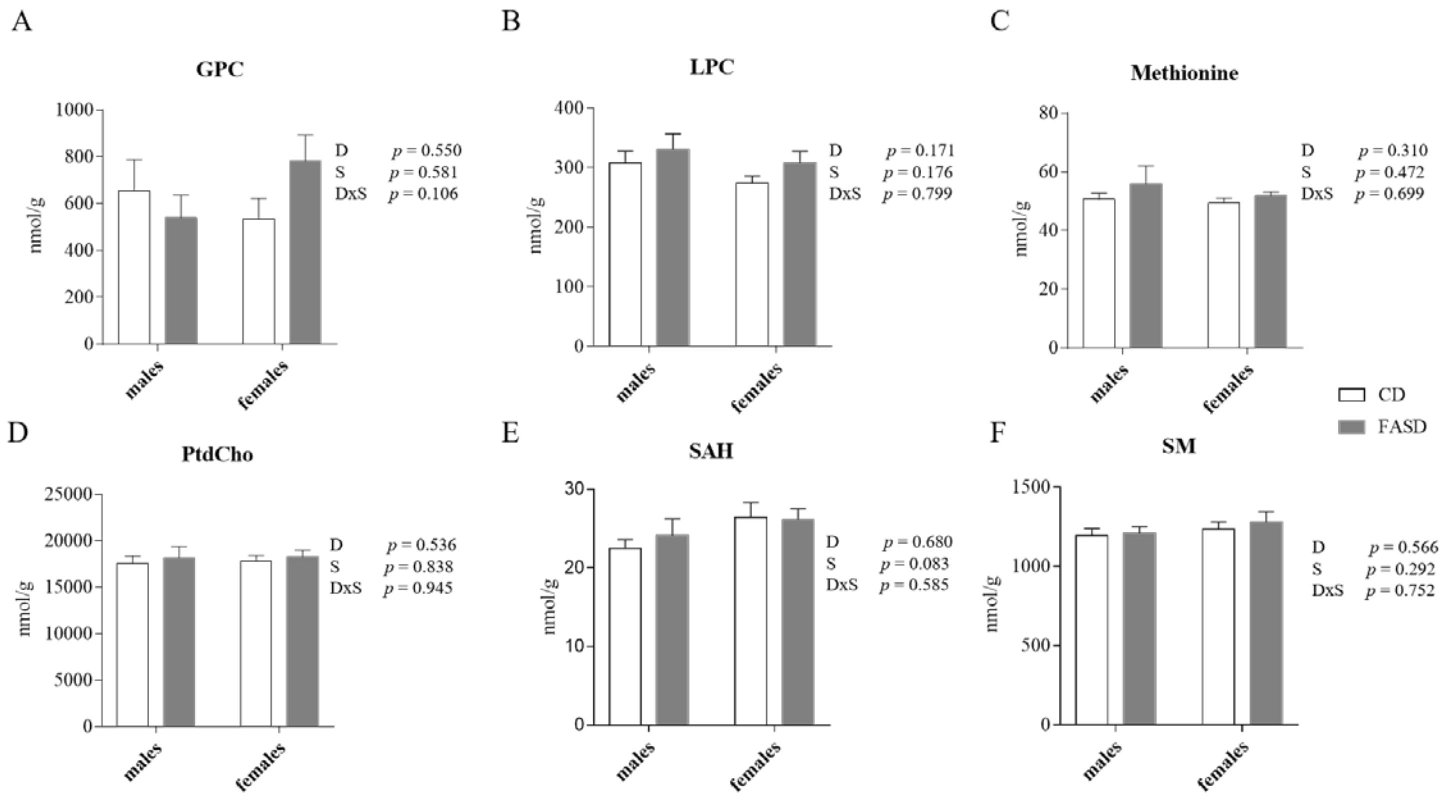

Figure S2. Concentrations of choline-derived metabolites and SAH measured by LC-MS in offspring liver.

There were no differences in glycerophosphocholine (GPC), lysophosphatidylcholine (LPC), methionine, phosphatidylcholine (PtdCho), S-adenosylhomocysteine (SAH) or sphingomyelin (SM) between groups (A, B, C, D, E, F, respectively). N=7-8/group. White bars: CD animals, gray bars: FASD animals. Values represent means ± SEM. *p* values from 2-factor ANOVA are indicated at the top of each graph. CD: Control diet, FASD: Folic acid supplemented diet, D: Diet, S: Sex, DxS: Diet x Sex interaction.

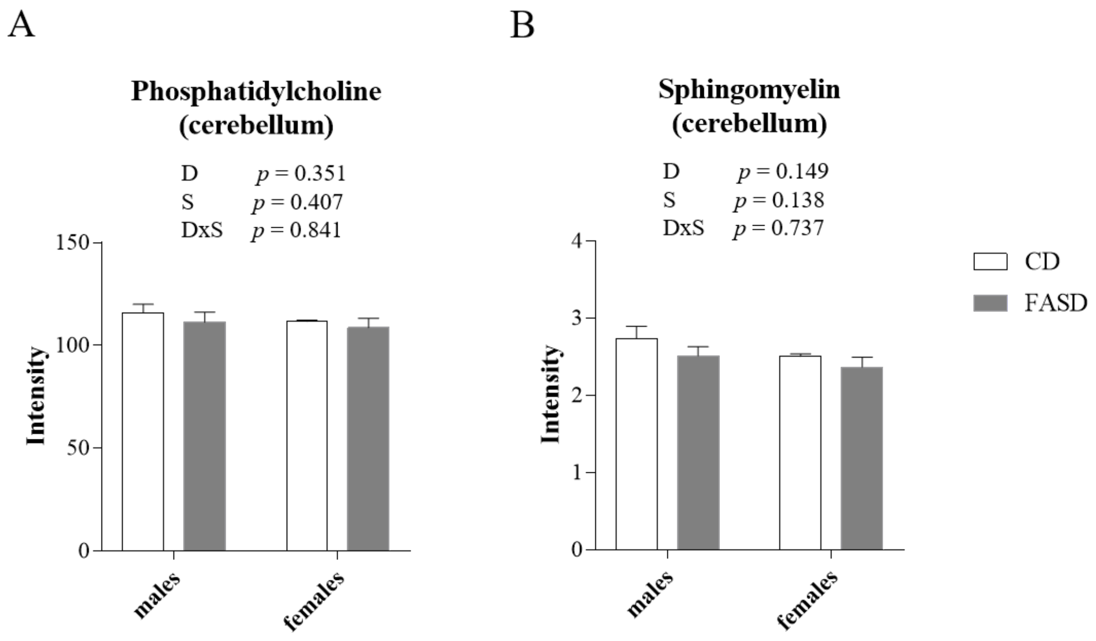

Figure S3. Phosphatidylcholine (PtdCho) and sphingomyelin (SM) measured by MALDI-IMS in offspring cerebellum.

No significant differences were detected in cerebellum (A) PtdCho (m/z 734+760) or (B) SM between FASD and CD pups. N=4/group. White bars: CD animals, gray bars: FASD animals. Bar graphs represent means ± SEM. *p* values from 2-factor ANOVA are indicated at the top of each graph. CD: Control diet, FASD: Folic acid supplemented diet, D: Diet, S: Sex, DxS: Diet x Sex interaction.
